# Correlations between intravoxel incoherent motion (IVIM) parameters and histological findings in rectal cancer: preliminary results

**DOI:** 10.18632/oncotarget.15753

**Published:** 2017-02-27

**Authors:** Alexey Surov, Hans Jonas Meyer, Anne-Kathrin Höhn, Curd Behrmann, Andreas Wienke, Rolf Peter Spielmann, Nikita Garnov

**Affiliations:** ^1^ Department of Radiology, Martin Luther University Halle-Wittenberg, Halle, Germany; ^2^ Department of Diagnostic and Interventional Radiology, University of Leipzig, Leipzig, Germany; ^3^ Department of Pathology, University of Leipzig, Leipzig, Germany; ^4^ Institute of Medical Epidemiology, Biostatistics, and Informatics, Martin Luther University Halle-Wittenberg, Halle, Germany

**Keywords:** rectal cancer, diffusion weighted imaging, intravoxel incoherent motion, histopathology, tumor grading

## Abstract

Our purpose was to correlate different intravoxel incoherent motion (IVIM), histopathological and clinical parameters in rectal cancer. 17 patients with histologically proven rectal cancer investigated on a 3.T device were included into the study. DWI was performed using a multi-slice single-shot echo-planar imaging sequence with b values of 0, 50, 200, 500 and 1000 s/mm.^2^ A polygonal region of interest was drawn within the tumors on every b image. The following parameters were retrieved from IVIM: apparent diffusion coefficient (ADC), true diffusion (D), pseudo diffusion coefficient (D*), perfusion factor (f), and relative perfusion f·D*. In every case, cell count, nucleic areas, proliferation index KI 67, and microvessel density were estimated on histopathological specimens. Pearson's correlation coefficient was used to analyze the association between the parameters. ADC correlated well with KI 67 index and D tended to correlate with cell count and KI 67. ADC and D tended to correlate with total nucleic area. The perfusion factor f correlated well with stained vessel area, total vessel area, and vessel count. D* and fD* correlated with mean vessel diameter. Distant metastasized tumors had higher D* and fD* values. IVIM parameter reflected different clinical and histopathological features in rectal cancer.

## INTRODUCTION

Diffusion weighted imaging (DWI) reveals motion of water molecules in biological tissue [1, 2]. The rate of diffusion in cellular tissues is described by apparent diffusion coefficient (ADC), which depends on different histological parameters, such as the number of membrane barriers that a diffusing water molecule encounters in a specified time interval [1–4]. Therefore, ADC reflects microstructure of investigated biological tissues [4–7]. Furthermore, according to the literature, ADC can be used to differentiate malignant from benign tumors [8, 9].

Some studies indicated that benign lesions had statistically significant higher ADC values than malignant tumors [8–10]. So Wang et al. showed these relationships for lesions in the head and neck region [11].

Similar results were reported for the colorectal region. For example, Jia et al. performed a meta analysis of the literature regarding DWI findings in several colorectal lesions [12]. The authors identified that the ADC values of malignant lesions varied from 0.97 mm^2^/s to 1.19 mm^2^/s, and benign lesions had higher ADC values ranging from 1.37 mm^2^/s to 2.69 mm^2^/s [12]. Furthermore, DWI can be superior in differentiating of malignant from benign bowel lesions in comparison to other sequences [12, 13]. So Busard et al. showed that only qualitative assessment of MR DWI may be valuable to facilitate differentiation between endometriosis infiltrating the bowel and colorectal carcinoma [13].

It has also been shown that DWI can provide information regarding aggressiveness of rectal cancer [14]. According to Curvo-Semedo et al., lower ADC values were associated with a more aggressive tumor profile [14]. Furthermore, significant correlations were found between mean ADC values and mesorectal fascia-status, N stage and differentiation grade of tumors [14].

Additionally, some reports mentioned that ADC can predict therapy success in rectal cancer. So Cai et al. revealed an increase in the mean tumor ADC during the course of neoadjuvant chemoradiation [15]. Moreover, a strong negative correlation between the mean pre-treatment tumor ADC and tumor regression after neoadjuvant chemoradiation was found [15]. Therefore, ADC can help predict or assess the response of rectal cancer to neoadjuvant chemoradiation at an early time point [15].

According to the literature, associations between DWI findings and clinical behavior of several tumors are related to relationships between DWI and histopathological parameters. It has been shown that ADC correlated well with proliferation index KI 67 in different lesions [5, 7, 16]. Furthermore, as reported previously, ADC also correlated inversely with cell count and nuclear areas [4, 6, 17].

Among ADC other parameters can be retrieved from DWI [18–20]. For example, intravoxel incoherent motion (IVIM) is an imaging technique for the separate estimation of tissue perfusion and diffusivity using multi-b-value DWI [18, 20]. It has been shown that perfusion fraction of IVIM reflected microvessel density in different tumors [19, 21, 22].

In rectal cancer, only few studies investigated associations between DWI findings and histopathology [23, 24]. Furthermore, the reported data were contradictory. For instance, Sun et al. found a significant correlation between ADC values and KI 67 level in rectal cancer [23]. Bäuerle et al., however, showed that IVIM parameters correlated with vascular density, but not with cellularity [24].

The purpose of the present study was to correlate different DWI, histopathological and clinical parameters in rectal cancer.

## RESULTS

The calculated IVIM parameters (mean values ± standard deviation) were as follows: ADC = 1.26 ± 0.36 × 10^−3^ mm^2^s^−1^; D = 1.07 ± 0.36 × 10^−3^ mm^2^s^−1^; f =20.47 ± 10.31%; D* = 257.62 ± 349.2 × 10^−3^ mm^2^s^−^1; fD* = 35.86 ± 48.92.

Analysis of histopathological specimens revealed the following results (mean values ± standard deviation): cell count = 1298.29 ± 358.31; total nucleic area = 155668.65 ± 41721.19 μm^2^; average nucleic area = 130.78 ± 42.59 μm^2^. The mean level of the proliferation index was 59.88 ± 28.93 %.

Analysis of CD 31 stained specimens identified the following parameters of microvessel density: stained vessel area = 22392.82 ± 19735.51 μm^2^; total vessel area = 27738.61 ± 24045.36 μm^2^; mean vessel diameter = 16.34 ± 7.15 μm; mean number of vessels = 75.06 ± 53.84.

As expected from their definition, ADC correlated well with D and D* correlated inversely with f and positively with fD* (Table 1). There were no further significant correlations between the DWI parameters.

**Table 1:** Correlations between different IVIM parameters

Parameters	D	D*	f	fD*	ADC
**D**	**x**	r=0.016 (p=0.951)	r=0.058 (p=0.824)	r=-0.044 (p=0.867)	**r=0.932 (p<0.0001)**
D*	**x**	**x**	**r=-0.498 (p=0.042)**	**r=0.923 (p<0.0001)**	r=-0.139 (p=0.595)
**f**	**x**	**x**		r=-0.369 (p=0.145)	r=0.403 (p=0.109)
fD*	**x**	**x**	**x**	**x**	r=-0.160 (p=0.539)

Significant correlations are highlighted in bold.

**Figure 1:**
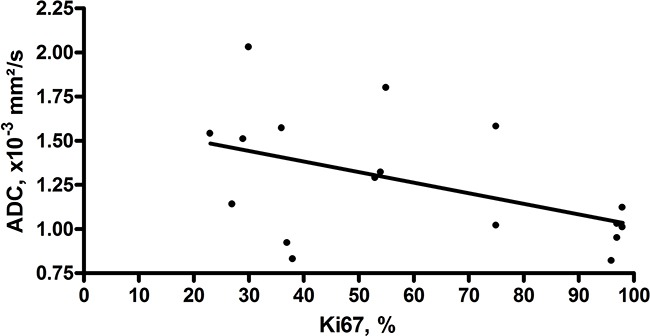
Correlation between ADC and KI 67 Correlation between ADC and KI 67. Correlation analysis shows a moderate inverse correlation between ADC and KI 67 index (r=-0.486, p=0.048).

Furthermore, correlation analysis between the identified IVIM and histopathological parameters was performed. As shown in Table 2, ADC correlated statistically significant with KI 67 index (Figure 1) and D tended to correlate inversely with cell count and KI 67 (Table 2). Additionally, D and ADC had a tendency to correlate with total nucleic area (Table 2).

**Table 2:** Correlations between ADC, D and histopathological parameters

Parameters	ADC	D
**Cell count**	r=-0.334 (p=0.190)	r=-0.449 (p=0.070)
**Total nucleic area**	r=-0.453 (p=0.068)	r=-0.463 (p=0.061)
**Average nucleic area**	r=0.059 (p=0.823)	r=0.161 (p=0.538)
**KI 67**	**r=-0.486 (p=0.048)**	r=-0.433 (p=0.082)

Significant correlations are highlighted in bold.

**Figure 2:**
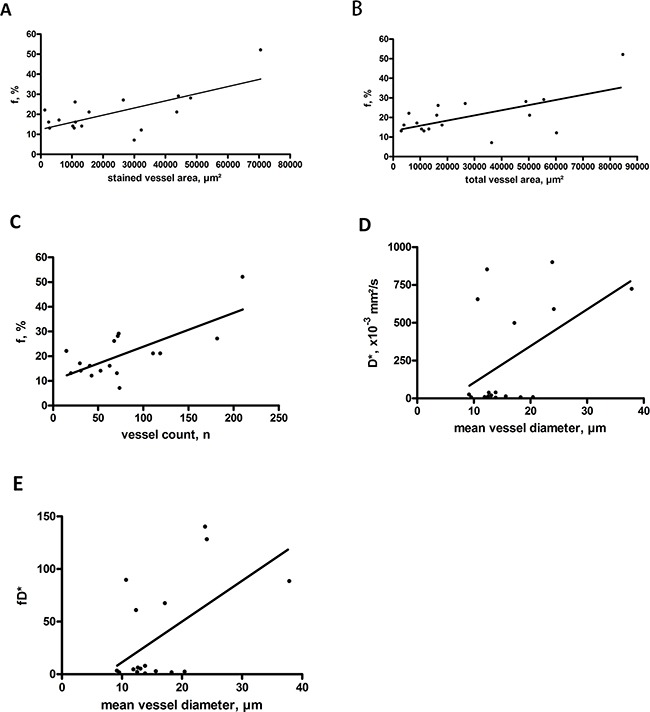
Associations between IVIM perfusion parameters and microvessel density in rectal cancer **(A)** Correlation between f and stained vessel area (r=0.682, p=0.003). **(B)** Correlation between f and total vessel area (r=0.614, p=0.009). **(C)** Correlation between f and vessel count (r=0.716, p=0.001). **(D)** Correlation between D* and mean vessel diameter (r=0.496, p=0.043). **(E)** Correlation between fD* and mean vessel diameter (r=0.567, p=0.018).

Correlation analysis between IVIM perfusion parameters and microvessel density showed the following results: the perfusion factor f had statistically significant correlations with stained vessel area, total vessel area, and vessel count (Figures 2A-2C) but not with mean vessel diameter (Table 3). On the contrary, D* and fD* correlated well with mean vessel diameter but not with other parameters of microvessel density (Figures 2D, 2E).

**Table 3:** Correlations between IVIM perfusion parameters and microvessel density

Parameters	Stained vessel area	Total vessel area	Vessel count	Mean vessel diameter
**f**, %	**r=0.682 (p=0.003)**	**r=0.614 (p=0.009)**	**r=0.716 (p=0.001)**	r=-0.154 (p=0.556)
D*	r=-0.185 (p=0.476)	r=-0.064 (p=0.806)	r=-0.353 (p=0.165)	**r=0.496 (p=0.043)**
fD*	r=-0.274 (p=0.287)	r=-0.155 (p=0.554)	r=-0.392 (p=0.119)	**r=0.567 (p=0.018)**

Significant correlations are highlighted in bold.

No significant differences were identified in DWI parameters between poorly and moderately differentiated tumors (Table 4).

**Table 4:** Comparison of IVIM parameters between moderately and poorly differentiated rectal cancer (RC)

DWI parameters	Moderately differentiated RC	Poorly differentiated RC	p values
**ADC**, × 10^−3^ mm^2^s^−1^	1.30 ± 0.38	1.07 ± 0.30	p=0.26
**D**, × 10^−3^ mm^2^s^−1^	1.14 ± 0.40	0.88 ± 0.24	p=0.20
**f**, %	18.35 ± 7.22	19.37 ± 6.78	p=0.80
D*, × 10^−3^ mm^2^s^−1^	281.20 ± 354.57	132.07 ± 255.78	p=0.42
fD*	32.41 ± 38.84	28.26 ± 55.82	p=0.87

The values are mean ± standard deviation.

**Figure 3:**
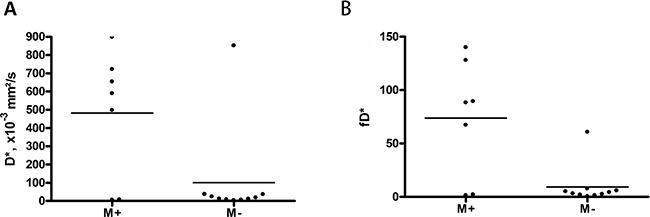
Comparison of perfusion IVIM parameters in rectal cancer with distant metastases (M+) vs tumors without distant metastases (M-) Tumors with distant metastases shows statistically significant higher D* (**A**) and fD* (**B**) values.

In addition, we compared DWI parameters in patients with distant metastases with those in patients without distant metastatic lesions. Distant metastasized tumors had higher D* and fD* values (Figures 3A, 3B), but there were no significant differences between other IVIM parameters (Table 5).

**Table 5:** Comparison of IVIM parameters in rectal cancer with distant metastases (M+) vs tumors without distant metastases (M-)

DWI parameters	M+	M-	p values
**ADC**, **× 10^−3^ mm^2^s^−1^**	**1.34 ± 0.37**	**1.21 ± 0.35**	**p=0.48**
**D**, × 10^−3^ mm^2^s^−1^	1.17 ± 0.36	0.99 ± 0.35	p=0.33
**f, %**	18.84 ± 6.77	21.55 ± 12.41	p=0.61
D*, × 10^−3^ mm^2^s^−1^	481.98 ± 347.63	100.57 ± 263.94	**p=0.021**
fD*	73.80 ± 55.06	9.31 ± 18.19	**p=0.021**

The values are mean ± standard deviation. Significant correlations are highlighted in bold.

## DISCUSSION

The present study revealed significant associations between different DWI and clinical and histopathological parameters in rectal cancer.

Some previous reports analyzed relationships between DWI as well as tumor grade in rectal cancer [14, 25, 26]. So Curvo-Semedo et al. found that ADC values correlated with differentiating grade of rectal cancer and tumor aggressiveness factors, such as N-stage and infiltration of the mesorectal fascia [14]. However, other researchers did not identify any correlations [23, 7]. For instance, Attenberger et al. could not found any associations between ADC and T-or N-stage of rectal cancer [25]. In the study of Akashi et al. there were no significant correlations between ADC values and T-stage, N-stage, mesorectal fascia status, and presence of lymphangiovascular invasion, but poorly differentiated carcinomas had lower ADC values in comparison to well and moderately differentiated tumors [26].

Although DWI has been reported as method which improved the sensitivity of T- and N-staging in rectal cancer [27], the local tumor expansion can also be well evaluated without DWI [26]. However, it is very important to predict if tumors develop systemic metastases (M stage) or not. In our study, we compared DWI parameters in tumors with distant metastases versus rectal cancer without systemic metastatic spread. To our best of knowledge, such analysis has not been performed previously. As seen, tumors with distant metastases had higher D* and fD* values in comparison to tumors without distant metastatic lesions. There were no significant differences in other DWI parameters between metastasized and non-metastasized carcinomas. Our findings suggest that tumor perfusion, i.e. microvessel density may play a significant role in the pathogenesis of metastases in rectal cancer. This is in agreement with some previous histopathological investigations [28, 29]. For instance, Takebayashy et al. found that microvessel counts from patients with lymph node metastasis, lymphatic vessel invasion, venous vessel invasion, or relapse were significantly higher than from those without [29]. Moreover, microvessel count was an independent prognostic factor in patients with rectal cancer according to the multivariate Cox hazard analysis [29]. In addition, Bognar et al. showed that tumor vascularization was linked to poor prognosis in overall survival in patients with rectal cancer and recurrence of liver metastases [30].

According to the literature, DWI parameters, especially ADC, are associated with the cell density of the investigated tissues [4, 7]. These relationships were documented in sarcomas, meningiomas, and several epithelial tumors [7, 16, 31]. In rectal cancer, only few studies investigated correlations between DWI and histopathological findings [23, 24, 32, 33]. Most of them were experimental studies. For example, in the study of Li et al. correlations between ADC value and pathologic indicies of colorectal tumor homografts in Balb/c mouse were analyzed [32]. The authors identified significant associations between ADC and apoptotic index p53 and proliferation index KI 67 [32]. Similar results were reported by Zhang et al. in the same tumor model [33]. Some clinical investigations also showed significant correlations between ADC and histopathology in rectal cancer [23, 24, 34]. So Heijmen et al. identified strong correlations between ADC values and KI 67 in liver metastases of colorectal cancer [34]. Furthermore, ADC values also correlated well with cell density [34].

According to Sun et al., also in primary tumors ADC values were associated with several histopathological parameters [23]. In particular, the authors found significant correlations between ADC and KI 67 [23].

In previous reports, only ADC values were retrieved from DWI [23, 24, 32, 33]. However, it is well known that DWI can also provide other significant parameters [18, 20]. As mentioned above, intravoxel incoherent motion (IVIM) provides beside ADC also different perfusion parameters [18–20]. Previously, only two studies analyzed relationships between IVIM parameters and histopathology in rectal cancer [21, 24]. Lee et al. studied IVIM data in an animal model of rectal cancer and found significant correlations between D*, f and vessel count [21]. Furthermore, Bäuerle et al. analyzed IVIM parameters in patients with rectal cancer [24]. The authors identified significant correlations between perfusion fraction f and vascular area fraction and between f and vascular diameter [24]. No significant correlations between DWI parameters and tumor cellularity were found [24].

In the present study, several associations between DWI and histopathology were detected. As seen, ADC correlated moderately with KI 67 and D tended to correlate with cell count and total nucleic areas. Perfusion parameters retrieved from DWI showed significant correlations with microvessel density. Interestingly, while the perfusion fraction f correlated well with stained and total vessel areas and with vessel count, but not with mean vessel diameter, D* and fD* correlated significantly with mean vessel diameter and not with stained and total vessel areas or vessel count. Overall, our findings suggest that IVIM parameters reflect different histopathological features in rectal cancer. Moreover, IVIM data were more sensitive in detection of these associations than ADC alone. Furthermore, our results indicate that neither ADC nor D, i.e. DWI marker for tumor cellularity and proliferation potential can be used to predict distant metastazation of rectal carcinomas. This confirmed indirectly previous histopathological analyses, which found that KI 67 level was not associated with the TNM stage of the colorectal adenocarcinoma [35].

Our study has several limitations. First, a small number of patients/tumors was investigated. Second, IVIM values were obtained from one ROI containing the largest available tumor, which might not be fully representative of the overall tumor profile.

Further studies with more patients are needed to confirm our findings.

In conclusion, different significant correlations between IVIM and histopathological parameters in rectal cancer were identified. Tumors with distant metastases showed statistically significantly higher D* and fD* values.

## MATERIALS AND METHODS

This is a IRB-approved study with prospectively acquired data and retrospectively analysis of them.

### Patients

Overall, 17 patients with histologically proven rectal cancer were included into the study. One patient was female and 16 were male with a mean age of 68.65 years (median age, 71 years; range, 51-76 years). In most cases, moderately differentiated adenocarcinoma was diagnosed (Table 6).

**Table 6:** Characteristics of tumors

Pathological diagnosis	n (%)
Well differentiated adenocarcinoma	2 (12)
Moderately differentiated adenocarcinoma	10 (59)
Poorly differentiated adenocarcinoma	5 (29)
**T stage**	
T2	3 (18)
T3	13 (76)
T4	1 (6)
**N stage**	
N0	4 (23)
N1	10 (59)
N2	3 (18)
**M stage**	
M0	10 (59)
M1	7 (41)

### Imaging

In all patients MRI of the pelvis was performed by using a 3.0 T device (Magnetom Skyra, Siemens, Erlangen, Germany). The imaging protocol included the following sequences: axial and sagittal T2 weighted (T2w) fat-supressed (fs) short tau inversion recovery (STIR) images, axial T2w turbo spin echo images, axial T1 weighted turbo spin echo (T1w TSE) images, and axial T1w TSE images with fat suppression after intravenous application of contrast medium (gadopentate dimeglumine, Magnevist, Bayer Schering Pharma, Leverkusen, Germany), in a dosis of 0.1 ml per kilogram of body weight. DWI was performed using a multi-slice single-shot echo-planar imaging (EPI) sequence with b values of 0, 50, 200, 500 and 1000 s/mm.^2^ The sequence parameters are summarized in Table 7.

**Table 7:** Sequences used in the study

Sequences	TR, ms	TE, ms	Matrix	FOV, mm	Slice thickness, mm
T1 TSE	661	9,8	400×400	410×512	5
T1 TSE	700	18	399×399	307×384	6
T2 TIRM	4000	34	399×399	269×384	5
T2 TSE	4000	108	280×280	365×384	3
T2 TIRM	5700	86	399×399	365×384	3
T1 TSE FS	941	10	280×280	652×768	3
T2 TSE	5000	65	399×399	614×768	3
T1 VIBE FS	4	1.8	380×380	480×640	2
EPI 2D	6700	58	307×400	83×130	5

TR – repetition time, TE – echo time, FOV – field of view, TSE – turbo spin echo, TIRM – turbo inversion recovery magnitude, EPI – echo planar imaging, VIBE - volumetric interpolated breath-hold examination.

All images were analyzed by one radiologist (A.S., 12 years radiological experience) on a PACS workstation (Centricity PACS, GE Medical Systems, Milwaukee, Wis., USA).

### DWI analysis

**Figure 4:**
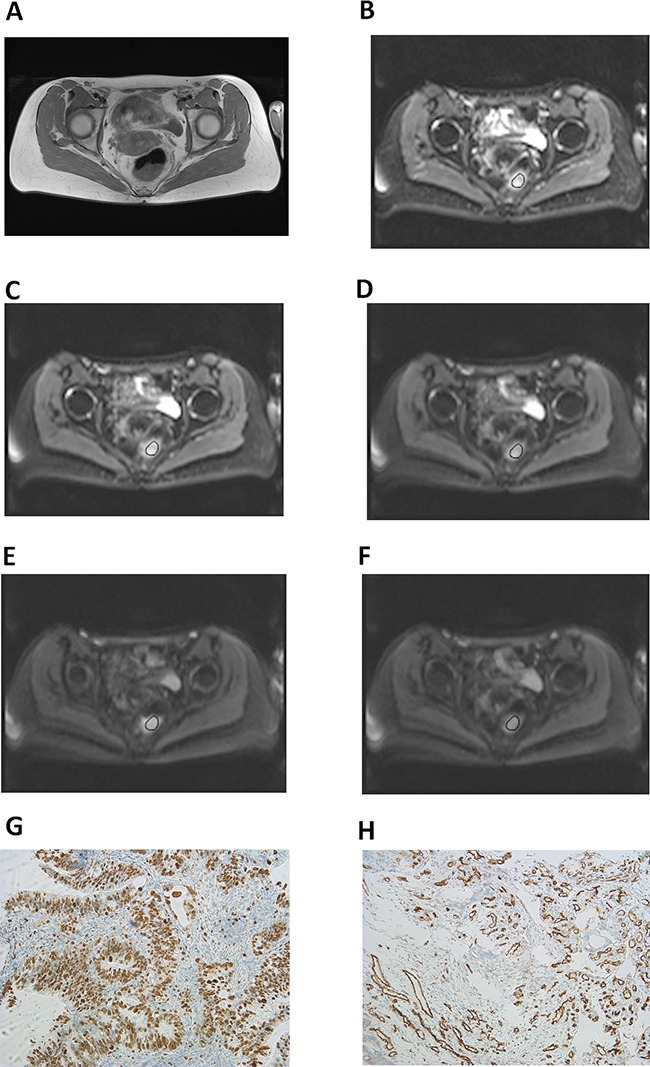
Imaging and histopathological findings in rectal cancer **(A)** T2w image showing a large rectal mass. Histopathological investigation after endoscopic biopsy confirmed the diagnosis of a moderately differentiated adenocarcinoma. Tumor stage: T3 N1 M0. **(B-F)** DW imaging findings: b0 (B), b50 (C), b200 (D), b500 (E), and b1000 (F). Estimated IVIM parameters are as follows: ADC=1. 02 × 10^−3^ mm^2^s^−1^, D=0.71 × 10^−3^ mm^2^s^−1^, f=28%, D*=16.60 × 10^−3^ mm^2^s^−1^, and fD*=5.03. **(G)** Immunohistochemical stain (MIB-1 monoclonal antibody). Histopathological parameters are as follows: Ki 67 index=75%, cell count=1390, total nucleic area= 225375.10 μm^2^, and average nucleic area=162.23 μm^2^. **(H)** Immunohistochemical stain (CD 31monoclonal antibody). Estimated microvessel parameters are as follows: stained vessel area=70555.98 μm^2^, total vessel area=84787.53 μm^2^, mean vessel diameter=11.97 μm, and vessel count=210.

The axial slices with the largest diameter of tumors were selected for estimation of signal intensities on b images. A polygonal region of interest (ROI) as large as possible was drawn around the margin of the tumors on b0 images (Figure 4). The position of the ROI was automatically placed also on all b images and on T2w, and pre- and postcontrast T1w images.

The mean diffusion weighted (DW) signal intensity values from the ROIs were analyzed using custom-made tool developed in MATLAB (MathWorks, Natick, MA) based upon the Levenberg–Marquardt fitting algorithm. According to the intravoxel incoherent motion (IVIM) theory [18], the DW signal was evaluated by using the following equation:

*SI*/*SI*_0_ = (1 − *f*) × exp(−*bD*) + *f* × exp(−bD*)  [1]

Where *SI* and *SI_0_* are the signal intensity at given b-values and at b=0 s/mm^2^, respectively; *D* and D* are the true diffusion and the pseudo diffusion coefficient, respectively; *f* is the fractional volume of capillary blood flowing in each voxel (i.e., perfusion factor).

Similar to the procedure described in Ref. 19, a two-step analysis was used to derive the diffusion parameters. First, true diffusion coefficient *D* was calculated by a mono-exponential function *SI*/*SI*_0_ = exp(−*bD*) using b-values larger than 200 s/mm^2^. Then, the D* and *f* coefficients were computed using eq. [1] for all b-values considering the calculated *D* values. The relative perfusion f·D* was also recorded [20].

### Histopathological analysis

In all cases the diagnosis of rectal cancer was confirmed histopathologically by endoscopic rectal biopsy. Representative tumor tissue slides from formalin-fixed paraffin-embedded tissue were processed after deparaffinization. The specimens were stained with MIB-1 monoclonal antibody and with CD31 antigen (both from DakoCytomation, Denmark) [24, 25]. All stained samples were digitalized by using a research microscope Jenalumar (Zeiss, Jena, Germany), with camera diagnostic instruments 4.2., magnification x400. Furthermore, the digital histopathological images were transferred as uncompressed TIFF images to ImageJ software (version 1.48v, NIH, Bethesda, MD) with a Windows operating system [17, 36]. Proliferation index KI 67, cell count, and microvessel density were estimated by using the program.

Proliferation index (KI 67) was calculated as percentage of stained nuclei on theMIB-1 stained specimens as reported previously [37]. The area with the highest number of positive tumor nuclei was selected for the analysis (Figure 4). Cell count was defined as a number of all nuclei on the MIB-1 stained specimens. Microvessel density included the following parameters: stained vessel area (μm^2^), calculated as CD 31 positive area divided by the total area of the analyzed histological specimens; total vessel area (μm^2^), i.e. a sum of stained vessel area and vessel lumen; total number of vessels (n); and, finally, mean vessel diameter (μm). In every case, all histopathological parameters were estimated per two high power fields a 0.16 mm^2^.

### Statistical analysis

For statistical analysis the SPSS statistical software package was used (SPSS 20, SPSS Inc., Chicago IL, USA). Collected data were evaluated by means of descriptive statistics (absolute and relative frequencies). Categorical variables were expressed as percentages. Analyses of DWI parameters were performed by means of two sided Mann-Whitney-U-tests. P-values < 0.05 were taken to indicate statistical significance in all instances. Pearson's correlation coefficient was used to analyze the association between IVIM and histological parameters.
